# Anti-Cancer Potential of Isoflavone-Enriched Fraction from Traditional Thai Fermented Soybean against Hela Cervical Cancer Cells

**DOI:** 10.3390/ijms25179277

**Published:** 2024-08-27

**Authors:** Amonnat Sukhamwang, Sirinada Inthanon, Pornngarm Dejkriengkraikul, Tistaya Semangoen, Supachai Yodkeeree

**Affiliations:** 1 Department of Biochemistry, Faculty of Medicine, Chiang Mai University, Chiang Mai 50200, Thailand; amonnat_s@cmu.ac.th (A.S.); sirinada_i@cmu.ac.th (S.I.); pornngarm.d@cmu.ac.th (P.D.); 2 Anticarcinogenesis and Apoptosis Research Cluster, Faculty of Medicine, Chiang Mai University, Chiang Mai 50200, Thailand; 3 Department of Medical Technology, Faculty of Allied Health Sciences, Burapha University, Chonburi 20131, Thailand; tistaya@go.buu.ac.th

**Keywords:** fermented soybean, genistein, daidzein, human cervical carcinoma cells, apoptosis, invasion, STAT3, MAPKs

## Abstract

Cervical cancer is a leading cause of gynecological malignancies and cancer-related deaths among women worldwide. This study investigates the anti-cancer activity of Thua Nao, a Thai fermented soybean, against HeLa cervical carcinoma cells, and explores its underlying mechanisms. Our findings reveal that the ethyl acetate fraction of Thua Nao (TN-EA) exhibits strong anti-cancer potential against HeLa cells. High-performance liquid chromatography (HPLC) analysis identified genistein and daidzein as the major isoflavones in TN-EA responsible for its anti-cancer activity. TN-EA and genistein reduced cell proliferation and induced G2/M phase arrest, while daidzein induced G1 arrest. These responses were associated with the downregulation of cell cycle regulators, including Cyclin B1, cycle 25C (Cdc25C), and phosphorylated cyclin-dependent kinase 1 (CDK-1), and the upregulation of the cell cycle inhibitor p21. Moreover, TN-EA and its active isoflavones promoted apoptosis in HeLa cells through the intrinsic pathway, evidenced by increased levels of cleaved Poly (ADP-ribose) polymerase (PARP) and caspase-3, loss of mitochondrial membrane potential, and the downregulation of anti-apoptotic proteins B-cell leukemia/lymphoma 2 (Bcl-2), B-cell lymphoma-extra-large (Bcl-xL), cellular inhibitor of apoptosis proteins 1 (cIAP), and survivin. Additionally, TN-EA and its active isoflavones effectively reduced cell invasion and migration by downregulating extracellular matrix degradation enzymes, including Membrane type 1-matrix metalloproteinase (MT1-MMP), urokinase-type plasminogen activator (uPA), and urokinase-type plasminogen activator receptor (uPAR), and reduced the levels of the mesenchymal marker N-cadherin. At the molecular level, TN-EA suppressed STAT3 activation via the regulation of JNK and Erk1/2 signaling pathways, leading to reduced proliferation and invasion of HeLa cells.

## 1. Introduction

Cervical cancer is the third most common type of cancer in women worldwide. In 2021, the cervical cancer incidence rate in Thai women was 13.8%, with a mortality rate of 6.8 deaths per 100,000 women [1]. Several treatment options for cervical cancer have been used, including radiation therapy, surgery, and chemotherapy. Platinum-base chemotherapy is frequently used in metastasis cervical cancer. Unfortunately, unpleasant side effects have been reported following long-term exposure to chemotherapeutic drugs. Hence, the development of natural ingredients from plant-based functional foods with minimal side effects and high efficacy for cancer treatment is of interest. Evidence from epidemiological studies have suggested that a high consumption of fermented soybean in Asian countries is related to a low incidence of breast and prostate cancer [2,3]. Therefore, fermented soybeans show promise in the development of functional foods with potential anti-cancer properties.

Thua Nao is a Thai fermented soybean that has been popularly used as a seasoning in northern Thai cuisine. Thua Nao is produced through the fermentation of boiled soybeans, utilizing a mixture of natural microflora. Fermented soybeans not only have high nutritional value but also exhibit various pharmacological activities, including antioxidant, anti-diabetic, anti-aging, anti-inflammatory, and anti-cancer properties [4,5]. Numerous bioactive compounds have been found in fermented soybeans, including peptides, phenolics, flavonoids, isoflavones, and saponins. Notably, isoflavones and peptides emerge as the principal functional constituents of fermented soybeans. Genistein and daidzein are major isoflavone aglycones present in fermented soybeans [6]. Both genistein and daidzein have demonstrated significant anti-cancer effects in animal studies, including against breast, prostate cancers, colon, liver, and cervical cancer. Interestingly, the epidemiological studies indicated an inverse relationship between soy consumption and breast cancer incidence. Clinical studies have shown that women with higher soy intake, which correlates with higher genistein levels, have a reduced risk of developing breast cancer Moreover, clinical trials have shown that genistein can reduce prostate-specific antigen (PSA) levels in patients with prostate cancer, suggesting a potential therapeutic role. Consequently, the contents and compositions of isoflavones in fermented soybeans are critical factors in determining their biological effects in humans.

Both genistein and daidzein have demonstrated significant anti-cancer effects in animal studies, including against breast, prostate, colon, liver, and cervical cancers [7,8,9]. Epidemiological studies have provided evidence for a protective role of isoflavones against the development of numerous cancers including breast cancer, cervical cancer, and prostate cancer. Clinical studies further support this, with evidence indicating that women with higher soy intake, which correlates with higher genistein and daidzein levels, have a reduced risk of developing breast cancer [10,11]. Additionally, clinical trials have demonstrated that genistein can lower prostate-specific antigen levels in patients with prostate cancer, highlighting its potential therapeutic role [12]. Therefore, the content and composition of isoflavones in fermented soybeans are crucial factors in determining their biological effects in humans.

The quality and composition of isoflavone in fermented soybean is dependent on the processing method, production process, and micro-organisms. The production process of Thua Nao is like Korean cheonggukjang and Japanese natto, which involve three main steps: soaking, boiling, and fermenting [13]. Previous studies have indicated the dominant bacterial genera found in Thua Nao are *Bacillus*, followed by *Lactobacillus*, *Enterococcus*, and *Globicatella* [14]. In many studies, Thua Nao has emerged as a functional food with antioxidant and anti-inflammatory properties [15]. Although, the anti-cancer effects of fermented soybeans have been extensively studied [16], unfortunately the molecular mechanism underlying the anti-cancer property of Thua Nao extract on human cervical carcinoma cells has not been elucidated.

In the current study, Thua Nao extract fractions were prepared by using different natural organic solvents. The HeLa human cervical carcinoma cells were treated with Thua Nao extract fractions and isoflavone aglycones. After treatment, cell viability and invasion were assayed. Additionally, we explored the mechanism of the active Thua Nao extract fraction and isoflavone on cell proliferation and apoptosis by examining the expression of cell cycle, proliferation, and apoptosis proteins. Moreover, we investigated the expression of proteins associated with metastasis. Furthermore, we determined the effects of Thua Nao on the signaling pathways involved in HeLa cell progression.

## 2. Results

### 2.1. Genistein and Daidzein in Thua Nao Ethyl Acetate Fraction (TN-EA)-Induced Human Cervical Carcinoma Cell (HeLa) Death

**Figure 1:**
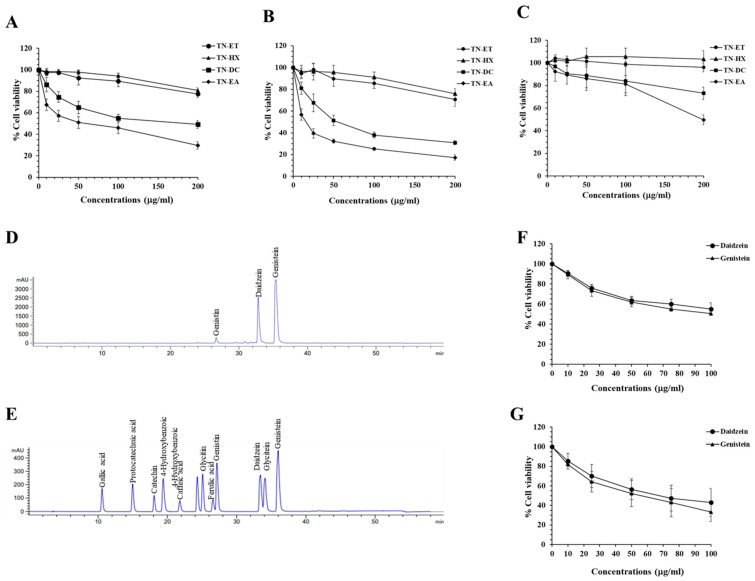
Cytotoxic effects of Thua Nao extract fractions and their active isoflavones in HeLa cells. HeLa cells were seeded into 96-well plates and treated with different Thua Nao extract fractions for 48 h (**A**) and 72 h (**B**). Cell viability was determined using the SRB assay. The cytotoxic effects of Thua Nao extract fractions on human skin fibroblasts were evaluated using the SRB assay after 72 h of treatment (**C**). HPLC chromatograms of TN-EA (**D**) and standard compounds (**E**) were obtained using an Eclipse Plus C18 column (5 μm, 4.6 × 250 mm), with an injection volume of 10 μL and UV-vis detection at 254 nm. The effects of the isoflavones genistein and daidzein on the viability of HeLa cells were measured at 48 h (**F**) and 72 h (**G**) after treatment. Data are expressed as the mean ± S.D. of three independent experiments.

To evaluate the anti-cancer activity of Thua Nao extract fractions on HeLa cells, the cells were treated with various concentrations (0–200 μg/mL) of the extract fractions for 48 and 72 h. Results from the SRB assay demonstrated that treatment with the Thua Nao ethyl acetate extract fraction (TN-EA) and the Thua Nao dichloromethane extract fraction (TN-DC) reduced HeLa cell viability, with IC_50_ values of 53 and 175 μg/mL, respectively, at 48 h, and 14 and 55 μg/mL, respectively, at 72 h. Conversely, treatment with the Thua Nao ethanolic extract fraction (TN-ET) and the Thua Nao hexane extract fraction (TN-HX) at 200 μg/mL reduced cell viability to 70.6% and 76.9%, respectively (Figure 1A,B). Moreover, Thua Nao extract fractions at 100 μg/mL induced cytotoxicity to fibroblast cells of less than 20% (Figure 1C). These results indicate that the TN-EA fraction displayed the most potent induction of human cervical cancer cell death without cytotoxicity to the normal fibroblast cell. Therefore, the TN-EA fraction was selected to identify the active compounds responsible for its anti-cancer activity. The HPLC chromatogram of TN-EA exhibited peaks corresponding to phenolic and flavonoid compounds with retention times matching those of the following standard compounds: genistin, daidzein, and genistein, as shown in Figure 1D,E. The actual contents of isoflavone in TN-EA are shown in Table 1. The main isoflavones in TN-EA were genistein, daidzein, and genistin, with the concentrations of 59.03, 57.17, and 8.70 μg/mg extract, respectively. Subsequently, we investigated the role of the main isoflavone in TN-EA fraction, genistein, and daidzein on HeLa cell survival. As shown in Figure 1F,G, treatment with genistein and daidzein reduced cell viability, with IC_50_ values of 100 μM and >100 μM, respectively, at 48 h, and 52 μM and 70 μM, respectively, at 72 h. The resulting data indicate that genistein and daidzein are the major active compounds that potentially serve to induce HeLa cell death in TN-EA fractions.

### 2.2. Anti-Proliferative Activity of TN-EA and Its Active Compounds on HeLa Cells 

**Figure 2:**
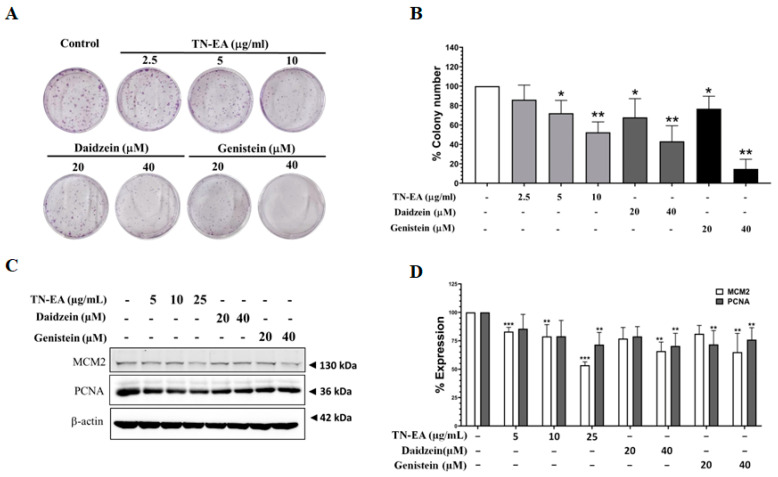
Anti-proliferative effects of TN-EA and active isoflavones on HeLa cells. (**A**) HeLa cells were exposed to TN-EA (0–10 μg/mL) and isoflavones (20 and 40 μM) for 48 h. After treatment, the compounds were withdrawn and the cells were cultured in DMEM with 10% FBS for 7 days. Proliferation was assessed using a colony formation assay. (**B**) Representative bar graph from the colony assay. (**C**) Effects of TN-EA, genistein, and daidzein on the expression of proliferative proteins were detected by Western blot. (**D**) Quantitative representation of protein marker levels (MCM2 and PCNA) normalized to β-actin. Data are mean ± S.D. of three independent experiments. * *p* < 0.05, ** *p* < 0.01, *** *p* < 0.001 compared to control.

**Figure 3:**
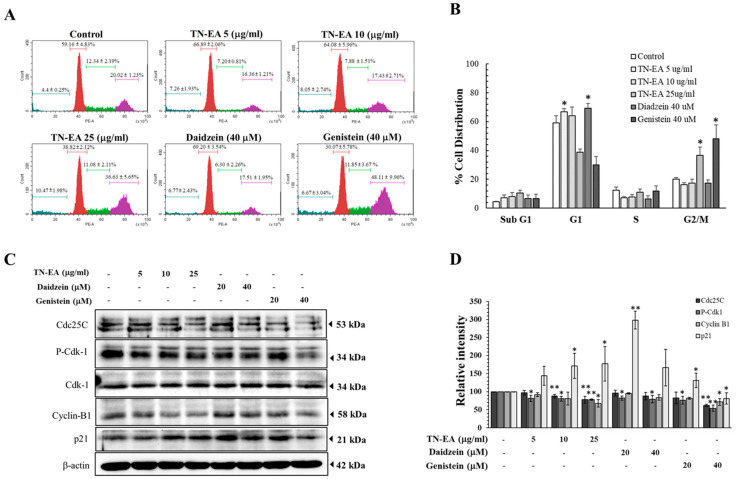
Effect of TN-EA, genistein, and daidzein on the cell cycle distribution of HeLa cells. HeLa cells were treated with TN-EA and its isoflavones for 24 h. (**A**) Cell cycle distribution was analyzed by PI staining and flow cytometry. (**B**) Data are presented as a bar graph. (**C**) Expression of cell cycle regulatory proteins (Cdc25C, phospho-Cdk1, Cdk1, cyclin B1, and p21) was detected by Western blot. (**D**) Quantification of protein expression is shown as a histogram. Data are mean ± S.D. of three independent experiments. * *p* < 0.05, ** *p* < 0.01 compared to control.

To assess the potential reduction in HeLa cell proliferation by TN-EA, genistein, and daidzein, the cells were treated with different concentrations of TN-EA and its active compounds for 48 h, and cell proliferation was determined by colony formation assay. As shown in Figure 2A,B, TN-EA, genistein, and daidzein significantly reduced HeLa cell colony formation. Moreover, the expression of proliferation proteins was investigated. The results presented in Figure 2C,D indicate that the protein levels of MCM2 and PCNA were reduced by TN-EA, genistein, and daidzein in HeLa cells. Next, the effect of TN-EA and its active compounds on the cell cycle modulation was determined by flow cytometer. Our data revealed that TN-EA at 25 μg/mL and genistein at 40 μM significantly induced HeLa cell cycle arrest in the G2/M phase. On other hand, daidzein at 40 μM remarkably elevated the percentage of HeLa cells in the G1 phase (Figure 3A,B). To elucidate the molecular mechanisms underlying TN-EA and its active compounds’ ability to induce cell cycle arrest, the expression levels of key cell cycle regulatory proteins were analyzed by Western blotting. As shown in Figure 3C,D, treatment with TN-EA and genistein resulted in decreased expression levels of Cdc25C, phospho-Cdk-1, and cyclin B1. However, daidzein at 40 μM reduced the expression level of phospho-Cdk-1 but did not affect Cdc25C and cyclin B1 levels. In contrast, TN-EA at 25 μg/mL, daidzein at 20 μM, and genistein at 20 μM significantly increased the expression of p21 in HeLa cells. These results indicate that TN-EA, daidzein, and genistein effectively induce cell cycle arrest by modulating the expression of critical cell cycle regulatory proteins. 

### 2.3. TN-EA, Daidzein, and Genistein Promote the Apoptosis of HeLa Cells

**Figure 4:**
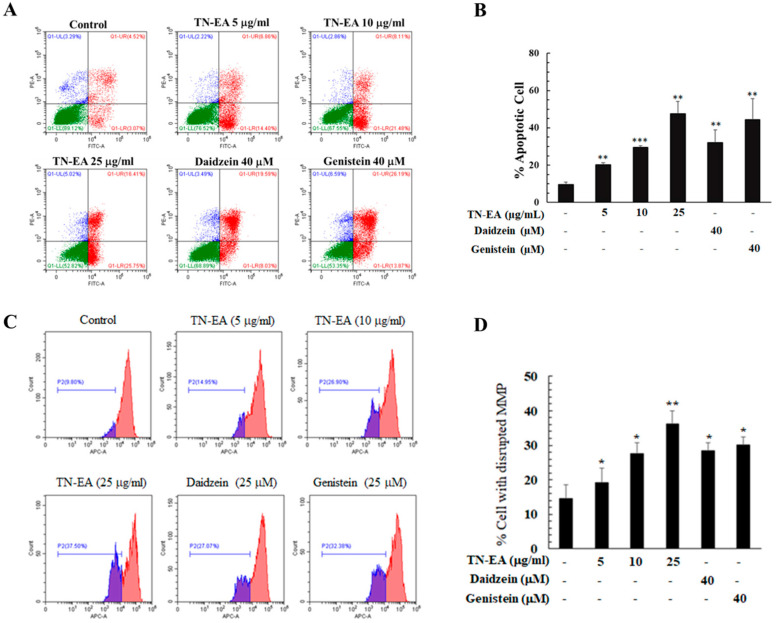
TN-EA, genistein, and daidzein induce apoptosis and disrupt mitochondrial membrane potential (MMP) in HeLa cells. (**A**) HeLa cells were treated with TN-EA and its active isoflavones for 48 h at indicated concentrations. After treatment, cells were stained with Annexin V-FITC and PI, then analyzed by flow cytometry. (**B**) Total apoptotic cells presented as a bar graph. (**C**) MMP alterations were investigated using MitoView™ 633 fluorescence staining and analyzed by flow cytometry. (**D**) Data presented as a bar graph. Data are mean ± S.D. of three independent experiments. * *p* < 0.05, ** *p* < 0.01, *** *p* < 0.001 compared to control.

To confirm whether TN-EA and its active compounds induce HeLa cell death through apoptosis, cells were stained with Annexin V-FITC and PI to observe the apoptotic population. As shown in Figure 4A,B, TN-EA significantly increased the apoptotic population in a dose-dependent manner compared to the control. Similarly, treatment with daidzein and genistein at 40 μM increased the apoptotic cell population to 33.0% and 46.1%, respectively. Mitochondrial dysfunction is a critical aspect of the apoptotic pathway, with the loss of mitochondrial membrane potential (MMP) serving as an indicator of intrinsic apoptosis. MMP alterations were investigated using MitoView™ 633 fluorescence staining, a dye that becomes brightly fluorescent upon accumulation in the mitochondria. Changes in MMP were analyzed by flow cytometry. The results showed that TN-EA significantly disrupted MMP in a dose-dependent manner, and both daidzein and genistein at 40 μM significantly increased the rate of MMP disruption (Figure 4C,D). 

**Figure 5:**
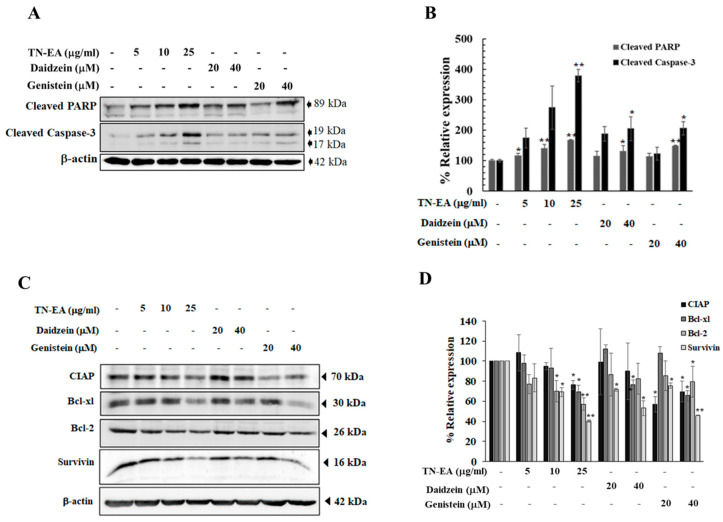
Effect of TN-EA and active isoflavones on apoptotic and anti-apoptotic protein expression in HeLa cells. HeLa cells were treated with TN-EA, daidzein, and genistein at indicated concentrations for 36 h. (**A**) Expression levels of apoptosis-associated proteins (cleaved-PARP and cleaved-caspase-3) were analyzed by Western blot. (**B**) Densitometric and statistical analysis of apoptotic protein expression, normalized to β-actin. (**C**) Levels of anti-apoptotic proteins (cIAP, Bcl-xL, Bcl-2, and survivin) were analyzed by Western blot. (**D**) Band intensity quantified and presented as a histogram. Data are mean ± S.D. of three independent experiments. * *p* < 0.05, ** *p* < 0.01 compared to control.

Further investigation into the effect of TN-EA and its active compounds on apoptosis-related proteins, including caspase-3 and Poly (ADP-ribose) Polymerase (PARP), revealed that cleavage of caspase-3 and PARP, a prerequisite for the final stage of cell death, was increased when HeLa cells were treated with TN-EA, daidzein, and genistein for 48 h (Figure 5A,B). Moreover, the expression levels of anti-apoptotic proteins, including cIAP, Bcl-2, Bcl-xL, and survivin, were reduced when cells were treated with TN-EA at 25 μg/mL and genistein at 40 μM. However, treatment with daidzein reduced the expression of Bcl-xL and surviving, but had no effect on cIAP and Bcl-2 (Figure 5C,D). These results demonstrate that TN-EA, daidzein, and genistein promote apoptosis in HeLa cells through the disruption of MMP and modulation of both pro- and anti-apoptotic proteins.

### 2.4. TN-EA, Daidzein, and Genistein Suppress the Migration and Invasion of HeLa Cells 

**Figure 6:**
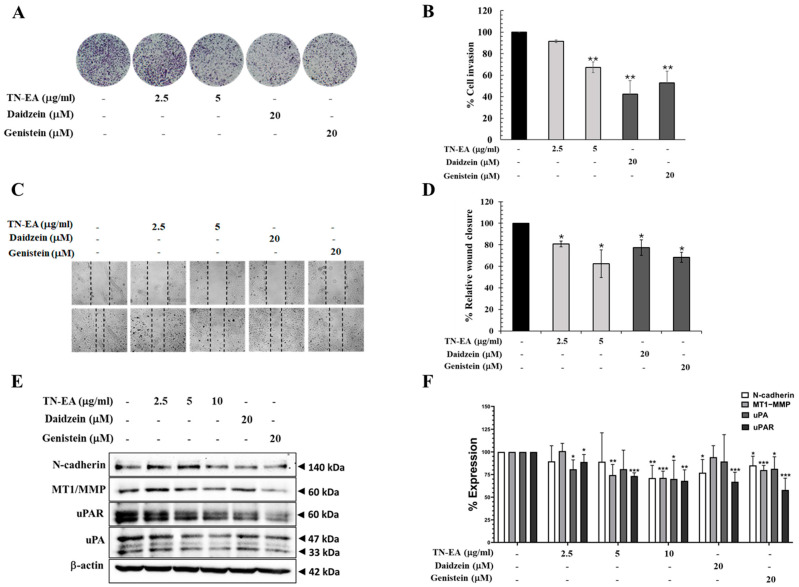
Anti-migration and invasion of TN-EA and active isoflavones. (**A**) Boyden chamber assay was performed to detect the effect of TN-EA, genistein, and daidzein on HeLa cell invasion, and (**B**) the percentage of cell invasion is presented in a bar graph. (**C**) Cell migration in HeLa cells was assessed after exposure to indicate concentration of TN-EA and isoflavones for 24 h using a wound-healing assay. (**D**) The bar graph represents the percentage of cell migration, quantified as the closure of the scratch area. (**E**) The expression levels of invasive proteins (N-cadherin, MT1-MMP, uPAR, and uPA) were analyzed by Western blot. (**F**) Densitometric and statistical analysis of invasive proteins expression, normalized to β-actin. Data are presented as mean ± S.D. values of three independent experiments. * *p* < 0.05, ** *p* < 0.01, and *** *p* < 0.001 compared to control.

To explore the effect of TN-EA and its active compounds on cell invasion, a Boyden chamber assay was conducted. As shown in Figure 6A,B, non-cytotoxic doses of TN-EA, daidzein, and genistein significantly prevented the invasion of HeLa cells through the Matrigel layer. Additionally, a wound-healing assay was performed to examine the migration of HeLa cells. After creating wounds by scratching, the cells were treated with TN-EA and its active compounds for 24 h. The results indicated that TN-EA, daidzein, and genistein significantly suppressed the migration of HeLa cells (Figure 6C,D). Alteration of the integrity between cancer cells and the extracellular matrix (ECM) is an important factor leading to cancer metastasis. Therefore, the effect of TN-EA, daidzein, and genistein on the expression of ECM degradation enzymes was investigated. Western blot analysis revealed that TN-EA and genistein significantly reduced the expression of MT1-MMP, uPA, and uPAR. Conversely, daidzein reduced the expression of uPAR but not MT1-MMP and uPA. Moreover, TN-EA, daidzein, and genistein reduced the levels of N-cadherin, a mesenchymal marker involved in the epithelial-mesenchymal transition (EMT) process (Figure 6E,F). These findings suggest that TN-EA and its active compounds, daidzein and genistein, inhibit HeLa cell invasion and migration through modulation of ECM degradation enzymes and suppression of N-cadherin expression. 

### 2.5. Effect of TN-EA, Daidzein, and Genistein on Oncogenic Signaling Pathways

**Figure 7:**
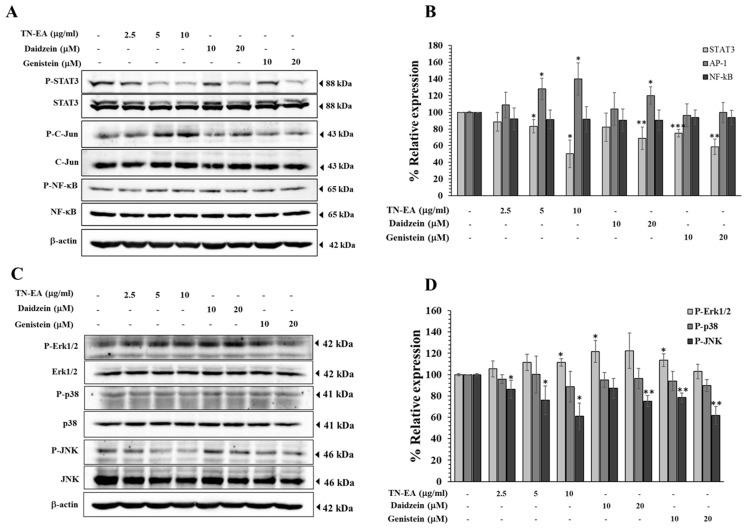
Modulation of oncogenic signaling pathways by TN-EA and active isoflavones. HeLa cells were treated with TN-EA, genistein, and daidzein for 24 h, and whole cell lysates were analyzed by Western blot. (**A**) Expression levels of phosphorylated and non-phosphorylated forms of oncogenic transcription factors (STAT3, c-Jun, and NF-kB) were determined. (**B**) Band density was quantified using ImageJ and presented as a histogram. (**C**) Levels of MAPK signaling proteins (Erk1/2, p38, and JNK) were detected. (**D**) Band intensity was quantified and presented as a histogram. Data are mean ± S.D. of three independent experiments. * *p* < 0.05, ** *p* < 0.01, *** *p* < 0.001 compared to control.

The activation of AP-1, NF-κB, and STAT3 transcription factors is known to be associated with proliferation and metastasis in human cervical cancer, primarily through the regulation of genes involved in cell survival, proliferation, and metastasis. Therefore, we investigated the effect of TN-EA and its active isoflavones on the phosphorylation of AP-1, NF-κB, and STAT3 using Western blot analysis. As shown in Figure 7A,B, treatment with TN-EA, daidzein, and genistein significantly decreased the phosphorylation of STAT3, whereas there was no significant effect on the phosphorylation of NF-κB. In contrast, TN-EA and daidzein stimulated the phosphorylation of c-Jun (AP-1), while genistein did not affect c-Jun phosphorylation. Mitogen-activated protein kinases (MAPKs) are a family of protein kinases involved in the progression of cervical cancer. Previous studies have reported an association between the activation of the MAPKs’ pathway and the regulation of STAT3 signaling. Therefore, we determined the effect of TN-EA and its active compounds on the activation of MAPKs, including Erk1/2, JNK, and p38, using Western blot analysis. The results showed that TN-EA, daidzein, and genistein significantly decreased the phosphorylation of JNK, while there was no significant effect on the phosphorylation of p38. Interestingly, TN-EA (10 μg/mL), daidzein (10 μM), and genistein (10 μM) increased the phosphorylation of Erk1/2 (Figure 7C,D). These findings suggest that the anti-cancer activity of TN-EA, daidzein, and genistein may involve the modulation of specific oncogenic signaling pathways, particularly through the inhibition of STAT3 and JNK phosphorylation.

## 3. Discussion

Cervical cancer is a major global health concern, being the most prevalent gynecological malignancy and the leading cause of cancer-related deaths among women worldwide. Survival rates vary significantly based on the stage of diagnosis. When detected early, the five-year survival rate can exceed 90%. However, in cases where the cancer has metastasized, the survival rate drops significantly [17]. Chemotherapy becomes the primary treatment option during metastasis, but it is associated with unpleasant side effects following long-term exposure to chemotherapeutic drugs. Many studies have shown that soybean fermentation products can inhibit cancer cell growth, induce apoptosis of cancer cells, and inhibit metastasis [18,19,20]. However, the anti-cancer properties of Thua Nao, a traditional Thai fermented soybean, on human cervical cancer cells have not been extensively investigated. Our findings demonstrate that the TN-EA fraction exhibits the highest anti-cancer potential against HeLa cells.

The anti-cancer properties of fermented soy products are primarily attributed to their isoflavone content. Aglycone isoflavones, specifically daidzein and genistein, are the major compounds found in fermented soybeans. These compounds are recognized for their broad-spectrum anti-cancer effects, which extend to various types of cancer including the breast, prostate, pancreas, lung, colon, and bone [9,21]. Our study identified genistein and daidzein as the major isoflavones in the TN-EA fraction. It is reasonable to hypothesize that the high concentrations of these main isoflavones in TN-EA likely contribute significantly to its observed anti-cancer activity. Additionally, our results showed that daidzein and genistein reduced the viability of HeLa cervical carcinoma cells, which is consistent with prior reports that these isoflavones are potent agents in decreasing the viability and proliferation of HeLa cells [21,22]. Beyond HeLa cells, genistein has also been reported to exhibit anti-cancer activity against various other types of cervical cancer cells, including CsSki, C33A, SiHa, and ME180 cells by inducing apoptosis and cell cycle arrest [23,24,25,26,27,28,29,30,31,32,33,34,35]. This suggests that the high levels of genistein and daidzein in the TN-EA fraction are likely responsible for its potent anti-cancer effects against human cervical cancer cells. 

We further elucidated the mechanisms by which TN-EA and its active isoflavones exert their anti-cancer effects by examining the proliferation of HeLa cells. Colony formation assays demonstrated a significant reduction in HeLa cell proliferation following treatment with TN-EA, genistein, and daidzein. However, the anti-proliferative effects of daidzein were relatively weak. These findings are consistent with previous reports indicating that genistein and daidzein reduce the proliferation of colon cancer cells, with genistein showing greater efficacy than daidzein. Moreover, treatments with TN-EA, genistein, and daidzein resulted in decreased expression of proliferation markers MCM-2 and PCNA, suggesting effective inhibition of cell proliferation. The cell cycle plays a crucial role in determining cell proliferation and regulates the complex processes governing cell growth and division. Numerous anti-cancer drugs have been demonstrated to arrest the cell cycle at specific phases, leading to inhibited cell proliferation. In our study, TN-EA and genistein significantly induced cell cycle arrest at the G2/M phase, while daidzein notably increased the percentage of HeLa cells in the G1 phase. These findings align with the report by Miller et al., which showed the capacity of genistein to induce cell cycle arrest in the G2/M phase and daidzein in the G1 phase in prostate cancer cells [26]. CDK1 proteins mainly control cell cycle progression, with CDK1 playing an essential role in the G2-to-M phase transition. The CDK1/Cyclin B1 complex is a key regulatory factor at the G2/M checkpoint. This complex is synthesized in large quantities as the cell passes through the G2/M phase. When G2/M cycle arrest occurs, the content of the CDK1/Cyclin B1 complex decreases correspondingly. The activation of the Cyclin B1/CDK1 complex is tightly regulated. Wee1 kinase phosphorylates and inhibits CDK1 to prevent premature entry into mitosis. Conversely, Cdc25C phosphatase removes these inhibitory phosphates to activate the Cyclin B1/CDK1 complex, allowing the cell to proceed into mitosis [27]. Moreover, p21 is a cyclin-dependent kinase inhibitor that plays a critical role in regulating the cell cycle by inhibiting the activity of cyclin-CDK complexes, thereby controlling cell cycle progression at multiple points, particularly at the G1 and G2/M phases. In the G1 phase, p21 can inhibit the activity of cyclin D/CDK4,6 and cyclin E/CDK2 complexes, while in the G2 phase it can inhibit the activity of the cyclin B/CDK1 complex [28]. Our study found that TN-EA and genistein reduced the expression of Cdc25C, phosphorylated CDK1, and Cyclin B1, while upregulating p21 levels, corresponding to G2/M phase arrest. This suggests that the TN-EA fraction and genistein effectively inhibit the transition from G2 to M phase by modulating these key regulatory proteins. In contrast, daidzein increased the level of p21 in HeLa cells but did not significantly affect the levels of Cdc25C and Cyclin B1. This indicates that daidzein induces G1 phase arrest by regulating p21 levels without significantly impacting the G2/M transition regulators. This finding suggests that genistein and daidzein may inhibit HeLa cell proliferation through different biological pathways.

Apoptosis is a crucial process in the cytotoxicity induced by anti-cancer agents, involving two primary pathways: the death receptor (extrinsic) and the mitochondrial (intrinsic) pathway. Numerous studies have reported that fermented soybean products induce apoptosis in various cancer cell lines, including gastric, colon, lung, and breast cancers, primarily through the intrinsic pathway [13]. Our data also demonstrate that TN-EA, daidzein, and genistein induce apoptosis in HeLa cells. The intrinsic pathway is often triggered by stress signals such as DNA damage or oxidative stress, leading to the release of cytochrome c from the mitochondria into the cytosol. In the cytoplasm, cytochrome c binds to Apaf-1 to form the apoptosome, which activates procaspase-9 into active caspase-9. Subsequently, caspase-9 activates caspase-3, a key executioner of apoptosis [29]. Caspase-3 cleaves various substrates, including PARP, a protein involved in DNA repair. The cleavage of PARP by caspase-3 ensures the irreversible progression of apoptosis. Consistent with apoptosis assays, our results indicate that TN-EA, daidzein, and genistein increase the levels of cleaved PARP and cleaved caspase-3, confirming their role in inducing apoptosis in HeLa cells. The perturbation of mitochondrial function has been observed to be required in intrinsic apoptotic cascade. Anti-cancer drugs may damage the mitochondria by increasing the permeability of the outer mitochondrial membrane, which is concomitant with the breakdown of the mitochondrial membrane potential (MMP) [30]. A drop in MMP disturbs intracellular ATP synthesis, the production of reactive oxygen species, the mitochondrial redox ratio, the translocation of cytochrome c to the cytosol, and induces apoptosis [31]. This pathway is precisely regulated by members of Bcl-2 family proteins. Bcl-2 and Bcl-xl are anti-apoptotic proteins that maintain mitochondrial integrity by preventing cytochrome c release, thereby inhibiting apoptosis [32]. Similarly, cIAP and survivin are anti-apoptotic proteins that inhibit caspase activity. Overexpression of Bcl-2, Bcl-xl, cIAP, and survivin is common in many cancers, which allows cells to evade the intrinsic apoptosis pathway [33]. This overexpression contributes to the resistance of cancer cells to programmed cell death. Anti-cancer drugs exert their effects by downregulating anti-apoptotic proteins such as Bcl-2, cIAP, and survivin, thereby promoting apoptosis [34]. Consistent with the above-mentioned studies, mitochondrial dysfunction was induced in TN-EA-, daidzein-, and genistein-treated cells, as confirmed by the loss of MMP, which was accompanied by modulation of the expression of pro-apoptotic proteins including Bcl-2, Bcl-xl, cIAP, and survivin. In agreement with our study, genistein and daidzein induced mitochondrial apoptosis in pancreatic and hepatic cancer cells via downregulation of the expression of Bcl-2 and Bcl-xl [35,36]. Taken together, our results suggest that TN-EA and its active isoflavones could induce HeLa cell apoptosis through the mitochondria-mediated apoptotic signaling pathway.

Cancer metastasis is a major cause of cancer mortality. Alteration of the integrity between cancer cells and the extracellular matrix (ECM) is an important factor leading to cancer metastasis. ECM degradation is crucial for metastasis, with enzymes such as matrix metalloproteinases (MMPs) and urokinase plasminogen activator (uPA) playing key roles [37]. Disruption of ECM degradation can inhibit invasion and reduce tumor metastasis. Our findings demonstrate that non-cytotoxic doses of TN-EA, genistein, and daidzein significantly reduce HeLa cell invasion and migration, correlating with decreased expression of MT1-MMP, uPA, and uPAR. Furthermore, EMT is vital for cancer invasion and metastasis [38]. During EMT, epithelial cells lose their polarity and adhesion, gaining migratory and invasive properties characteristic of mesenchymal cells. In the process of EMT, E-cadherin expression decreases while N-cadherin increases, promoting cell detachment and interaction with the extracellular matrix [39]. Our study shows that TN-EA, daidzein, and genistein reduce N-cadherin expression, consistent with previous reports indicating that genistein and daidzein inhibit breast and prostate cancer invasion by regulating MMP-9 expression and the EMT process [7]. These results suggest that TN-EA and its active isoflavones can effectively reduce HeLa cell invasion, at least partly through the modulation of ECM degradation enzymes and EMT marker expression. 

Genistein and daidzein exert their anti-cancer effects by targeting multiple signaling pathways involved in cancer cell growth, survival, and metastasis. For instance, genistein has been shown to suppress colon cancer cell growth by inhibiting the PI3K/Akt pathway [40]. Additionally, genistein has demonstrated anti-metastatic effects in cervical cancer cells by regulating the FAK-paxillin and MAPK signaling pathways [41]. These isoflavones also function as both ER agonists and antagonists in estrogen-sensitive tissues, including the breast, ovaries, and cervix. While it is generally believed that isoflavones inhibit the growth of ER-positive breast cancer cells and endometrial adenocarcinoma through their estrogen antagonistic properties, the mechanisms underlying their anti-cancer effects in other cancer types are still not fully understood [9,42]. Some studies suggest that isoflavones exert physiological actions that are either partially ER-dependent or entirely ER-independent, indicating the possibility of alternative pathways. Notably, Yamashita et al. found that equol and daidzein inhibited cell proliferation in HeLa cells, which express very low levels of ERα and ERβ, suggesting that these compounds may inhibit proliferation through an ER-independent mechanism [43].

STAT3, AP-1, and NF-κB are critical oncogenic transcription factors that play a crucial role in the carcinogenesis of various cancer cells, including cervical cancer [44,45]. These transcription factors regulate the expression of proteins associated with proliferation, survival, and metastasis [36]. Our study demonstrated that TN-EA, daidzein, and genistein decreased the activation of STAT3, while there was no significant effect on the activation of NF-κB. Consistent with our results, Liu et al. reported that genistein downregulated the expression of survivin and cyclin D1 through the inhibited phosphorylation of STAT3, leading to apoptosis in pancreatic cancer [46]. Constitutive STAT3 activation has been associated with various human cancers and has indicated poor prognosis, including in cervical cancer cells. Activation of STAT3 is involved in proliferation regulation by stimulating the expression of cell cycle regulators such as p21, cyclin D1, cyclin B, cyclin E1, Cdc25C, and c-Myc [47,48]. Conversely, inhibition of STAT3 results in the activation of apoptotic signaling pathways, as evidenced by the downregulation of anti-apoptotic proteins like Bcl-2, Bcl-xL, survivin, and cIAP. Additionally, STAT3 signaling pathways have been reported to stimulate cervical cancer cell metastasis by facilitating EMT progression and upregulating the expression of ECM degradation enzymes, including MT1-MMP, uPA, and uPAR [49]. Since the activation of AP-1 promotes cancer progression, inhibition of AP-1 has been shown to reduce cell proliferation and invasion [50]. Previous studies have shown that regulation of AP-1 by flavonoids is controversial. Genistein, biochanin kaempferol, and hesperitin quercetin induced AP-1 activity at low concentrations, while high concentrations inhibited AP-1 activity in prostate cancer [51]. Similarly, our findings indicated that TN-EA and daidzein at non-cytotoxic doses stimulated AP-1 activity, suggesting the presence of multiple signaling mechanisms. These studies support our findings, indicating that TN-EA and active isoflavones regulate HeLa cell survival, proliferation, and invasion by reducing the activation of the STAT3 signaling pathway.

The JAK/STAT3 pathway is crucial for activating STAT3 in response to pro-inflammatory cytokines like IL-6, IL-10, IL-17, and IL-20. STAT3 activation can also be modulated independently of the JAK/STAT3 pathway through crosstalk with MAPK signaling pathways [52]. The MAPK pathways, including Erk1/2, p38, and JNK, are implicated in cancer cell proliferation, survival, and metastasis. Previous studies have linked specific MAPK family members to the modulation of STAT3 signaling in various cell types [53,54]. For instance, STAT3 activity has been observed to be inhibited through Erk1/2- and p38-dependent pathways in human lung adenocarcinoma, while JNK inhibition reduces STAT3 serine phosphorylation in oral squamous cell carcinoma [55,56]. Similarly, Guo et al. found that JNK inhibition decreases STAT3 activation in breast cancer cells [57]. Our study showed that TN-EA, daidzein, and genistein significantly decreased JNK activation without affecting p38, while inducing Erk1/2 phosphorylation. These results suggest that TN-EA and its active isoflavones inhibit STAT3 activation at least in part by modulating the JNK and Erk1/2 MAPK signaling pathways.

The bioavailability of genistein and daidzein is essential for determining their biological activity. These isoflavones are primarily absorbed in the small intestine, with moderate absorption rates leading to peak plasma concentrations within 4–8 h post-ingestion, typically ranging from 0.1 to 5 μM [58]. Following absorption, both compounds undergo extensive phase II metabolism in the liver, where they are conjugated with glucuronic acid and sulfate, resulting in conjugated forms that dominate in the bloodstream [59]. The distribution of these isoflavones in tissues after ingestion plays a critical role in their bioactivity. Studies have shown that genistein can accumulate in breast tissue, where it may exert protective effects against breast cancer, with concentrations in breast tissue being lower than in plasma but still biologically active [60,61]. Additionally, Rannikko et al. reported that in prostate cancer patients, the levels of genistein and daidzein in prostate tissue increase following phytoestrogen consumption, suggesting that their biological functions could be exerted directly within the prostate [62]. A double-blind phase II clinical trial further supports the safety and efficacy of genistein in prostate cancer treatment, highlighting its potential therapeutic benefits [63]. Furthermore, before recommending the use of isoflavones in treating cervical cancer, additional studies are needed to investigate their distribution in cervical tissue and validate their effects in clinical human trials.

In summary, TN-EA fraction from Taou Nao exerts anti-cancer activity against HeLa cervical cancer cells. The efficacy of TN-EA is largely attributable to its high concentrations of genistein and daidzein, which demonstrate potent inhibitory effects on HeLa cell viability and proliferation. TN-EA and its isoflavones exert these effects by inducing cell cycle arrest at specific phases, and modulating key regulatory proteins. High doses of TN-EA and genistein induce cell cycle arrest at the G2/M phase, characterized by the downregulation of Cdc25C, phospho-Cdk-1, and cyclin B1. Conversely, high doses of daidzein markedly elevate the percentage of HeLa cells in the G1 phase, with a concurrent reduction in phospho-Cdk-1 expression. These findings suggest that the modulation of these key regulatory proteins by TN-EA and its constituents is critical to their ability to inhibit cell proliferation and promote cell cycle arrest in HeLa cells. Moreover, TN-EA and its constituents promote apoptosis via the mitochondrial pathway, as evidenced by disrupted mitochondrial membrane potential, enhanced cleavage of caspase-3 and PARP, and the downregulation of anti-apoptotic proteins such as Bcl-2 and survivin. Additionally, TN-EA effectively reduces HeLa cell invasion and migration by downregulating ECM degradation enzymes and EMT marker. Moreover, TN-EA inhibits the activation of STAT3 partially through the modulation of the JNK and Erk1/2 MAPK signaling pathways. These findings suggest that TN-EA and its active isoflavones hold promise as potential therapeutic agents for cervical cancer.

## 4. Materials and Methods

### 4.1. Chemicals and Reagents 

Dulbecco’s modified eagle’s medium (DMEM), penicillin/streptomycin, and trypsin-EDTA were purchased from Gibco (Grand Island, NY, USA). Fetal bovine serum (FBS) was supplied by Hyclone (Logan, UT, USA). An FITC Annexin V kit was obtained from Elabscience Biotechnology (Houston, TX, USA). Antibodies specific to Erk1/2, p-c-Jun, p-p65 (p-NF-κB), N-cadherin, Bcl-xl, Bcl-2, cIAP, survivin, and β-actin were obtained from Cell Signaling Technology (Danvers, MA, USA), whereas antibodies specific to p-ERK, p38, p-p38, STAT3, p-STAT3, c-Jun, MCM2, PCNA, Cylin B1, MT1-MMP, uPA, p-Cdk-1, Cdk1, Cdc25C, cleaved PARP, cleaved caspase-3, and p21 were purchased from Abclonal (Woburn, MA, USA). Antibodies for the detection of uPAR, and p65(NF-κB) were purchased from Santa Cruz Biotechnology (Santa Cruz, CA, USA). Nitrocellulose membrane and ECL reagent were supplied by GE Healthcare (Little Chalfont, UK). Matrigel was purchased from Becton Dickinson (Bedford, MA, USA). Standard genistein, daidzein, glycitein, genistin, diadzin, and glycitein, with purity of more than 98% was ordered from Chengdu Biopurify Phytochemicals Ltd. (Chengdu, China).

### 4.2. Preparation of Thua Nao Extract Fractions

Thua Nao was prepared by the traditional method. Briefly, 1 kg of soybean was soaked in water for 12 h. After soaking, the soybeans were subjected to boiling for a period of 3 h and allowed to cool down to 40 °C. The cooked soybeans were then subjected to a natural fermentation process for 3 days at 37 °C and 80% humidity. Thua Nao was air-dried at 50 °C by a hot air oven, and powdered. A ground powder (500 g) was extracted twice with 80% (*v*/*v*) ethanol overnight at room temperature. The solvent was then filtered, concentrated under vacuum evaporation, and freeze-dried to obtain the ethanolic fraction. The ethanolic fraction (TN-ET) was subsequently fractionated using different polarity of organic solvent including hexane, dichloromethane, and ethyl acetate, then concentrated under vacuum evaporation and air-dried to obtain the hexane (TN-HX), dichloromethane (TN-DC), and ethyl acetate (TN-EA) fraction. 

### 4.3. High-Performance Liquid Chromatography (HPLC) Analysis

The isoflavone and phenolic contents in TN-EA were determined by HPLC using an Eclipse Plus C18 (5 μm, 4.6 × 250 mm, Agilent, Santa Clara, CA, USA). The samples were dissolved in ethanol and injected 10 μL to HPLC with UV detection. The mobile phase system was methanol as solution A and 0.1% v/v trifluoroacetic acid (TFA) in water as solution B. The gradient used was: 0 min 100% of solution B and 50 min 100% of solution A. The fingerprint of samples was detected and compared with standard compounds found in fermented soybean including genistein, daidzein, glycitein, genistin, diadzin, glycitein, catechin, protocatechuic acid, and ferulic acid. The contents of each isoflavone were calculated by the peak area under the curve, compared with the standard calibration curve.

### 4.4. Cells and Cell Cultures 

The Hela cells were obtained from Elabscience (Cat. No. EP-CL-0101) and human skin fibroblast was obtained from the American type culture collection (ATCC, Manassas, VA, USA). Both cells were cultured in DMEM supplement with 10% fetal bovine serum (FBS) and 1% penicillin-streptomycin. All cell lines were maintained at 37 °C in 5% CO_2_ in a humidified environment. For Thua Nao extract fractions and isoflavones treatment, the compounds were dissolved in DMSO, and then diluted with the culture medium, ensuring that the final concentration of DMSO was less than 0.5% (*v*/*v*).

### 4.5. Cell Viability Assay

The cytotoxicity of Thua Nao extract fractions and isoflavones on HeLa and fibroblast cells was determined using the sulforhodamine B (SRB) assay. HeLa cells and fibroblast cells were plated at a density of 3.0 × 10^3^ cells/well in a 96-well plate and incubated at 37 °C in a 5% CO_2_ atmosphere overnight. They were then treated with various concentrations of Thua Nao extract fractions (0–200 µg/mL) or isoflavones (0–100 μM) for 48 and 72 h. At the end of the treatment period, 100 µL of 10% (*w*/*v*) trichloroacetic acid (TCA) was added to fix the cells, followed by incubation at 4 °C for 1 h. The plate was then washed with slow-running water, tapped on a paper towel to remove excess water, and allowed to air-dry at room temperature. Subsequently, the cells were stained with an SRB solution for 30 min and washed three times with 200 µL of 1% (*v*/*v*) acetic acid. The protein-bound dye was dissolved in 200 µL of a 10 mM Tris-base solution, and the absorbance was measured using a microplate reader at 510 nm.

### 4.6. Colony Formation

A colony formation assay was employed to examine the effect of TN-EA (0–10 μg/mL) or daidzein (20 and 40 μM) and genistein (20 and 40 μM) on HeLa cell proliferation. HeLa cells were plated at a density of 500 cells/well in a six-well plate and incubated at 37 °C in a 5% CO_2_ atmosphere overnight. The cells were treated with TN-EA, daidzein, or genistein for 48 h. After incubation with tested compounds, the culture media was removed and the cells continued to culture in drug-free DMEM for 7 days. The resulting colonies were fixed with glutaraldehyde (6.0% *v*/*v*) for 20 min. Subsequently, the cells were stained with crystal violet (0.5% *w*/*v*) for 45 min. After staining, the colonies were washed three times with deionized water, and colony counting was performed using an iBrightTM CL-1500 imaging system. Clusters with a cell count exceeding 100 were considered colonies.

### 4.7. Cell Cycle Assay

HeLa cells (1 × 10^5^ cells/well) were seeded into a 6-well plate and incubated at 37 °C in 5% CO_2_ overnight. The cells were treated with TN-EA, daidzein, or genistein in DMEM containing 10% FBS for 24 h. Following treatment, the cells were fixed with ice-cold 70% (*v*/*v*) ethanol for 30 min. Nuclear DNA content-staining was carried out by adding propidium iodide (PI) (50 µg/mL) and RNase A (25 µg/mL) in PBS, followed by incubation at 37 °C for 30 min in the dark. Cell cycle distribution was calculated after appropriate gating of cell populations in a FL-2-Area vs. FL-2-Width plot of PI fluorescence using a flow cytometer (Beckman Coulter DxFLEX). The data acquisition and analysis were done using using CytExpert for DxFLEX 2.0 software. 

### 4.8. Apoptosis Assay

The apoptosis of HeLa cells, after being treated with TN-EA, Daidzein, or Genistein, was determined by an Annexin V-FITC/PI Apoptosis Detection Kit FITC (Elabscience Biotechnology Inc., Houston, TX, USA). Briefly, HeLa cells were treated with tested compounds for 48 h. After treatment, cells were harvested with trypsin and stained with 2.5 µL of propidium iodide (PI) and 2.5 µL of Annexin V-FITC at room temperature for 15 min. The stained cells were analyzed using the flow cytometer. CytExpert for DxFLEX 2.0 software was used for the data analysis.

### 4.9. Mitochondrial Membrane Potential

Mitochondrial membrane potential was assessed using MitoViewTM 633 (Biotium, Fremont, CA, USA), according to the manufacturer’s protocol. Briefly, Hela cells were treated with TN-EA, daidzein, or genistein for 48 h. The cells were collected using trypsinization and incubated with 100 nM of MitoViewTM 633 in incomplete DMEM for 20 min in the dark at 37 °C in a humidified atmosphere containing 5% CO_2_. Then, the cells were washed with PBS, and fluorescence was recorded at excitation/emission of 638/660 nm by flow cytometry. Data were analyzed using CytExpert for DxFLEX software.

### 4.10. Wound-Healing Assay

To determine the effect of TN-EA, daidzein, and genistein on the migration of HeLa cells, a wound-healing assay was performed. HeLa cells (1 × 10^5^ cells/well) were seeded in a 12-well plate and cultured in DMEM containing 10% FBS overnight. When the cells reached 100% confluence, a straight line with the same width was scratched across the monolayer, and PBS was used to wash away non-adherent cells. After that, the cells were treated with TN-EA, daidzein, or genistein in DMEM with 0.5% FBS. Images were captured using a phase contrast microscope in 4 random fields at 0 and 24 h. The distance of wound closure was quantified according to the space of migrating tumor cells using ImageJ (v1.410).

### 4.11. Invasion Assay 

The invasion of the HeLa cells was investigated using the modified Boyden chamber method, as previously described. Briefly, polyvinylpyrrolidone-free polycarbonate filters (Millipore, Carrigtwohill, Tullagreen) with a pore size of 8 µm were coated with 50 µL of fibronectin (10 µg/mL) and Matrigel (10 µg/50 µL). HeLa cells were placed in the upper chamber at a density of 1 × 10^5^ cells with or without tested compounds. The medium in the lower chamber containing 10% FBS was added as a chemoattractant. The cells were then incubated for 18 h. After incubation, the cells that had invaded the lower surface of the membrane were fixed with methanol and stained with 1% (*w*/*v*) toluidine blue. The migrated cells on the lower surface of the filter were counted.

### 4.12. Western Blot Analysis 

The whole-cell lysate was then separated via SDS-PAGE electrophoresis and transferred to a nitrocellulose membrane via electroblotting. Following this, the membrane was blocked and incubated with primary antibodies in a one-step solution (Bio-Helix, New Taipei, Taiwan) for 2 h at room temperature. Subsequently, the membrane was incubated with a secondary antibody (diluted to 1:20,000) in a one-step solution for 1 h. Following incubation, the membrane was washed five times with PBS containing 5% tween for 5 min each, and then stored in PBS before imaging. For protein visualization, chemiluminescence-based development techniques were employed, and images were captured using the iBrightTM CL-1500 imaging system. The quantitative expression of each protein was determined by analyzing band density using ImageJ (v1.410).

### 4.13. Statistical Analysis 

All data were from at least three independent experiments. Statistical analysis was performed using the IBM^®^ SPSS^®^ Statistics V.28.0.1.0. Student’s *t*-test was applied to compare the different means. Mean differences were considered significant when * *p* < 0.05, ** *p* < 0.01, *** *p* < 0.001.

## Figures and Tables

**Table 1:** Determination of phenolic and flavonoids in TN-EA fraction by HPLC.

Standard	Concentration in TN-EA (μg/mg Extract)
Gallic acid	ND
Protocatechuic acid	ND
Catechin hydrate	ND
4-Hydroxybenzoic acid	ND
Caffeic acid	ND
Daidzin	ND
Glycitin	ND
Ferulic acid	ND
Genistin	8.702
Daidzein	57.172
Glycitein	ND
Genistein	59.035

ND, not detected.

## Data Availability

Data is contained within the article.
